# Thoracic Electrical Bioimpedance in Pregnancy: Applications During Pregnancy with an Emphasis on the Management of Hypertensive Disorders

**DOI:** 10.3390/jcm14238463

**Published:** 2025-11-28

**Authors:** Alfredo F. Gei, Nathalia Martínez Tobar, Gustavo Hernández Martínez, Thomas N. Bischoff Ogas

**Affiliations:** Houston Center for Maternal Fetal Medicine, 7900 Fannin Suite 4400, Houston, TX 77054, USA; alfredo.gei@gmail.com (A.F.G.); gustavo.hm098@gmail.com (G.H.M.); thomas.ogas@gmail.com (T.N.B.O.)

**Keywords:** thoracic electrical bioimpedance, impedance cardiography, non-invasive cardiography, hypertensive disorders of pregnancy, preeclampsia, maternal hemodynamics

## Abstract

**Background:** Hypertensive disorders of pregnancy (HDP), including gestational hypertension and preeclampsia, affect up to 10% of pregnancies worldwide and remain a leading cause of maternal and perinatal morbidity and mortality. These conditions are associated with adverse fetal outcomes, including preterm birth, growth restriction, and maternal complications such as stroke, eclampsia, multi-organ dysfunction, and a higher risk of long-term cardiovascular complications. Current management relies largely on intermittent blood pressure monitoring and assessment of symptoms, approaches that provide limited insight into the complex hemodynamic alterations underlying HDP. **Objective:** This narrative review aims to synthesize the available evidence on noninvasive cardiography through thoracic electrical bioimpedance (TEB) as a tool for maternal hemodynamic monitoring in pregnancy, with a focus on hypertensive disorders. Specifically, we (1) describe maternal cardiovascular adaptations in normal gestations and their disruption in HDP, (2) provide an overview of thoracic electrical bioimpedance cardiac output (TEBCO) technology, (3) summarize validation studies in pregnant populations, (4) explore potential clinical applications, including diagnostic support, therapeutic guidance, fluid management and postpartum surveillance, and (5) identify key limitations and research priorities for future practice. **Conclusions:** Noninvasive cardiography through thoracic electrical bio-impedance is an underutilized tool in the medical field. As an alternative to invasive assessment, TEBCO can identify underlying pathologic hemodynamic changes susceptible to treatment and allow monitoring of hemodynamic trends. The implementation of TEBCO would allow pathophysiologic-based treatments, improve clinical response to therapy, and lead to potential prolongations of pregnancy and cost-savings in healthcare. Current evidence is limited by small sample sizes, device variability, and lack of outcome-based trials. Future research should focus on standardized validation, multicenter studies, and interventional trials to determine whether non-invasive cardiography-guided care can improve maternal and neonatal outcomes.

## 1. Introduction

Hypertensive disorders of pregnancy (HDP), including gestational hypertension and preeclampsia, remain a major global health challenge. These conditions affect an estimated 5–10% of pregnancies worldwide and are responsible for a substantial proportion of maternal deaths, particularly in low- and middle-income countries [1,2]. In addition to maternal mortality, HDP are a leading cause of severe morbidity, including stroke, renal failure, hepatic dysfunction, and postpartum hemorrhage. From the fetal perspective, they are associated with intrauterine growth restriction, preterm birth, and perinatal mortality [3]. The burden of disease extends beyond the peripartum period, as women with a history of preeclampsia face a two- to fourfold increased risk of future hypertension, ischemic heart disease, and stroke [4].

Despite these significant short- and long-term consequences, clinical management of HDP continues to rely predominantly on intermittent blood pressure monitoring and symptom surveillance. While BP measurement is indispensable for diagnosis and surveillance, it provides a limited perspective on maternal cardiovascular status. Other measures, such as intra-arterial blood pressure monitoring, central venous and pulmonary artery catheters, provide a clearer hemodynamic picture of the pregnant woman, due to their invasive nature. However, these interventions are reserved for critically ill women in intensive care settings.

Normal pregnancy is characterized by significant cardiovascular adaptations, including expansion of plasma volume, increases in cardiac output, and reductions in systemic vascular resistance (SVR), all of which support uteroplacental perfusion and fetal growth [5,6]. In women who develop HDP, these adaptations are disrupted, but the underlying hemodynamic patterns are heterogeneous. Some patients exhibit elevated cardiac output and tachycardia which correspond to a “hyperdynamic” phenotype, whereas others display low cardiac output and high vascular resistance—a vasoconstrictive, “hypodynamic” phenotype [7,8]. Such variability may explain why antihypertensive therapies show inconsistent efficacy: a drug that reduces afterload may benefit a vasoconstricted patient, but compromise perfusion in one with a high output state [9].

These observations have prompted growing interest in methods that allow characterization and real time, noninvasive hemodynamic monitoring. Thoracic electrical bioimpedance (TEB), also known as impedance cardiography (ICG) or thoracic electrical bioimpedance cardiac output (TEBCO), is a technology capable of continuously measuring cardiac output, stroke volume, SVR, and thoracic fluid content through surface electrodes [10,11]. Other noninvasive methods of hemodynamic assessment include transthoracic echocardiography and focused cardiac ultrasound, both of which are capable of assessing cardiac output, ventricular function, volume status, and valvular abnormalities. Doppler ultrasound can be utilized for serial hemodynamic profiling and for fluid responsiveness assessment. Pulse wave velocity measures arterial stiffness, providing additional information about the vascular changes that occur during pregnancy [12].

While other noninvasive methods for hemodynamic measurements are capable of providing useful clinical data, one advantage TEB has over ultrasound-based methods and echocardiography is the fact that it is operator-independent and can easily provide continuous, real-time data. TEB devices also tend to be more accessible and less technically demanding than echocardiography or Doppler-based monitors.

By providing dynamic cardiovascular information without the risks of invasive monitoring, TEB offers a potentially valuable adjunct in obstetric care, particularly for conditions in which hemodynamic alterations are at the basis of their pathophysiology, such as hypertension or shock.

## 2. Methods

For purposes of this review, literature was identified through electronic searches of PubMed and Web of Science databases. Search terms included combinations of “thoracic electrical bioimpedance”, “impedance cardiography”, “cardiac output”, “pregnancy”, “preeclampsia”, “gestational hypertension”, and “hemodynamics”. The search included studies published in English between 1921 and 2025, with no restriction on study design. Additional articles were identified by screening reference lists of relevant publications. Both experimental and observational studies evaluating TEBCO and impedance-based methods in human pregnant or postpartum populations were included, with an emphasis on those addressing hypertensive disorders of pregnancy.

## 3. Hemodynamic Assessment in Hypertensive Disorders of Pregnancy

### 3.1. Maternal Hemodynamics in Normal Gestation and Hypertensive Disorders

From the first trimester, systemic vasodilation occurs as a result of increased production of nitric oxide, prostacyclin, and relaxin, along with reduced sensitivity to vasoconstrictors such as angiotensin II [13]. This vasodilatory state reduces systemic vascular resistance (SVR) by up to 30–35% by mid-gestation, leading to a fall in mean arterial pressure (MAP) despite concurrent rises in plasma volume and cardiac output (CO). Plasma volume itself expands by approximately 40–50% across pregnancy, driven by activation of the renin–angiotensin–aldosterone system and enhanced sodium and water retention [14,15]. Heart rate rises by 10–20 beats per minute, while stroke volume (SV) increases through improved myocardial compliance and enhanced contractility [16,17]. The net effect is a 30–50% rise in CO by the end of the second trimester, which helps ensure adequate uteroplacental perfusion and fetal oxygenation [16].

Structural adaptations complement these functional changes. Echocardiographic studies demonstrate mild, reversible eccentric hypertrophy of the left ventricle and enhanced diastolic filling, reflecting accommodation to increased preload and volume load [18].

In HDP, these physiological adjustments are often incomplete or maladaptive. Preeclampsia is characterized by abnormal placentation, systemic endothelial dysfunction, and excessive vasoconstriction, leading to hypertension and end-organ injury [3]. Women with preeclampsia frequently exhibit inadequate plasma volume expansion, increased SVR, and impaired diastolic relaxation [19,20].

Importantly, HDP does not represent a uniform hemodynamic state. Investigators have described at least two major phenotypes. In early-onset preeclampsia, typically associated with impaired placentation, women often present with a hypodynamic profile of reduced CO and markedly elevated SVR. Conversely, late-onset preeclampsia may be associated with a hyperdynamic phenotype, characterized by increased CO, increased heart rate, and relatively preserved SV [7,21]. Similar heterogeneity has been reported in gestational hypertension [21].

### 3.2. Clinical Relevance of Hemodynamic Heterogeneity

The heterogeneity of hemodynamic patterns in HDP has direct implications for management. Blood pressure measurements, while essential for diagnosis and monitoring, reflect only the product of CO and SVR, without differentiating the underlying circulatory elements [8]. As a result, two women with identical BP values may require different therapeutic strategies. For example, vasodilators such as hydralazine or calcium channel blockers may be beneficial for women with high SVR, whereas beta-blockers may be more effective in those with high output physiology [9,20]. In the absence of hemodynamic monitoring, such individualized treatment decisions are largely based on clinical judgment, previous experience, hospital policies and medication availability/familiarity, which in turn may contribute to variability in therapeutic responses.

Current guidelines for the management of acute hypertension emphasize the use of calcium channel blockers, or betablockers in the management of a hypertensive crisis, regardless of the mechanism and pathophysiologic basis of the hypertension. This vertical monotherapy might be inappropriate for many patients as the mechanism is neither known, nor purely hyperdynamic or vasoconstrictive in most cases [22].

Fluid management is similarly challenging in HDP. Although many women with preeclampsia appear volume depleted intravascularly, they may also have significant interstitial edema due to increased vascular permeability [23]. Empirical fluid resuscitation carries the risk of exacerbating pulmonary edema, one of the leading causes of maternal mortality in severe preeclampsia [24]. Conversely, under-resuscitation can worsen uteroplacental hypoperfusion and compromise fetal well-being. To date, no guidelines consider the use of volume or diuretics in the management of pregnant hypertensive women since their use has been considered potentially harmful to the respiratory status of the mother or to placental and fetal perfusion, respectively.

These risks underscore the need for accurate, noninvasive, and dynamic tools to assess maternal hemodynamics in real time.

## 4. Thoracic Electrical Bioimpedance (TEBCO) Technology

### 4.1. History/Development

The history of electrical bioimpedance research can be traced back more than a century. In the 1921 publication “*Les lois de la résistance électrique des tissus vivants*”, Philippson examined the impedance of blood cells and animal tissues across frequencies from 500 kHz to 3 MHz, anticipating modern studies of tissue frequency responses [25]. Earlier physiologists such as du Bois-Reymond had explored the electrical properties of biological tissues, but Philippson’s work, followed by the studies of Debye, Fricke, and Cole, provided the foundation for the field. In the mid-20th century, Nyboer, Geddes, and others advanced the clinical applications of impedance plethysmography for cardiovascular assessment, laying the groundwork for TEB [26].

Clinical applicability expanded in the latter half of the 20th century. Thomasset (1962) and Hoffer (1969) [27,28] demonstrated that whole-body impedance could estimate total body water, bridging theoretical work with clinical utility. In the early 1980s, Mills and colleagues applied impedance to monitor hydration in U.S. Navy personnel, leading RJL Systems to develop specialized devices. Soon after, Lukaski and colleagues (1985) published a seminal study showing that fat-free mass could be estimated from whole-body impedance, moving bioelectrical impedance analysis (BIA) into mainstream clinical and research use. The field has continued to expand with international conferences and the establishment of the *Journal of Electrical Bioimpedance* in 2010, highlighting its transformation from early physiological curiosity to a clinical tool with direct impact on patient care [26].

### 4.2. Basic Principles of Thoracic Electrical Bioimpedance

**Figure 1:**
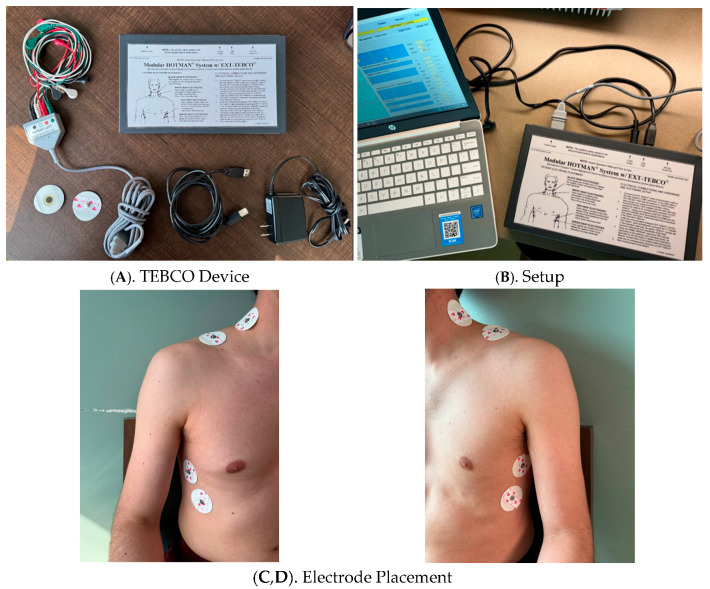
Thoracic Electrical Bioimpedance (TEBCO) Monitoring System and Electrode Placement.

The underlying principle of TEB is based on the relationship between electrical impedance and thoracic blood volume. A high-frequency, low alternating current is applied across the thorax using electrodes positioned at the base of the neck and along the lower thorax (Figure 1).

Due to the presence of electrolytes, blood is a good electrical conductor, and cyclical changes in blood volume and velocity with each heartbeat cause small but measurable fluctuations in thoracic impedance. These changes are captured in real time and processed by device-specific algorithms, which calculate stroke volume and, by extension, cardiac output [29,30].

Modern TEBCO devices refine this approach with bioreactance technology, which analyzes phase shifts in voltage rather than absolute impedance changes. This method is thought to improve signal-to-noise ratios and enhance measurement stability, particularly in patients with motion- or ventilation-related artifacts [31,32].

TEBCO generates a set of hemodynamic variables that are highly relevant to pregnancy and HDP:

- Cardiac Output (CO): The total volume of blood pumped by the heart per minute.
- Stroke Volume (SV): The amount of blood ejected with each ventricular contraction.
- Systemic Vascular Resistance (SVR): A derived measure of afterload, calculated from CO and mean arterial pressure.
- Thoracic Fluid Content (TFC): A surrogate marker of intrathoracic volume status, which increases with pulmonary congestion.
- Additional indices, including cardiac index (CI), velocity index, and left ventricular ejection time, provide complementary data [30].

**Figure 2:**
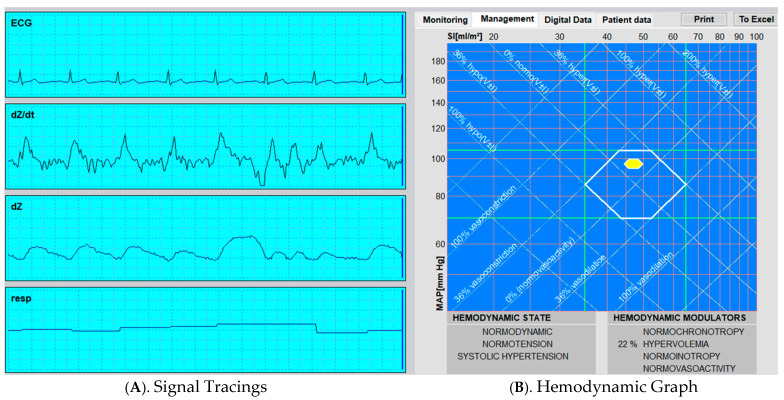
Representative Output from Thoracic Electrical Bioimpedance (TEBCO) Monitoring.

This data is displayed in a variety of ways depending on the model utilized (Figure 2).

### 4.3. Limitations of TEBCO

Despite the advantages, important limitations remain. Accuracy and reproducibility compared with gold-standard methods are variable. The technique estimates stroke volume and cardiac output from changes in thoracic impedance using algorithms (e.g., Kubicek or Sramek–Bernstein) that model the thorax as a uniform cylindrical volume conductor with fixed geometry. These assumptions are oversimplified in many clinical states such obesity, pleural effusions, lung edema, or marked changes in intrathoracic pressure, where thoracic anatomy and conductivity deviate substantially from the model, leading to biased stroke volume estimates [33]. While some studies report moderate to strong correlations between TEB and echocardiography or thermodilution, the limits of agreement are often wide. Technical challenges can also affect measurement reliability. Proper electrode placement is critical yet may be challenging in women with increased body mass index, significant breast tissue, or skin conditions that alter thoracic impedance [31]. Obesity represents another important challenge for thoracic electrical bioimpedance. In individuals with overweight and morbid obesity, excess adipose tissue alters thoracic geometry, reduces baseline thoracic impedance, and changes the distribution of intrathoracic blood volume, all of which reduce the validity of the cylindrical volume assumptions used in standard stroke volume equations. As baseline impedance falls, the magnitude of the signal can become disproportionately amplified relative to true aortic flow, leading to systematic overestimation of stroke volume and cardiac output [34]. Studies in bariatric surgery and intensive care unit populations have repeatedly demonstrated this effect. Bernstein et al. reported that standard impedance cardiography algorithms tended to overestimate stroke volume in patients with morbid obesity and emphasized that the Sramek–Bernstein weight adjustment factor does not correct this limitation. Similarly, Lagrand et al. found significantly reduced accuracy and wider limits of agreement for impedance-derived cardiac index measurements in ICU patients with morbid obesity compared with normal-weight controls, concluding that impedance cardiography should be used with caution in this group [35]. Woltjer et al. demonstrated that obesity significantly decreased the accuracy of impedance-derived stroke volume estimates, and they found no evidence to support the Sramek–Bernstein weight correction factor widely used in ICG algorithms [36]. Similarly, perioperative validation studies using electrical cardiometry reported that overweight and obesity were associated with greater bias and reduced precision in cardiac index measurements compared with reference techniques [37]. Obstetric data have raised important considerations regarding the interpretation of thoracic electrical bioimpedance during pregnancy. The physiologic changes accompanying gestation, including increased blood volume, altered thoracic fluid distribution, reduced baseline impedance and upward displacement of the diaphragm, modify several of the assumptions underlying classical impedance algorithms. In a longitudinal study of healthy pregnancies, Moertl et al. observed that progressive changes in thoracic fluid distribution and waveform morphology, particularly in late gestation, could lead to unreliable absolute stroke volume calculations [38]. The dynamic process of labor and delivery can introduce motion artifacts, reducing signal stability. Additionally, arrhythmias disrupt the detection of ventricular ejection time, thereby impairing stroke volume estimation. Different commercial platforms employ variable electrode spacing, proprietary algorithms, and signal-processing methods. As a result, inter-device variability can lead to divergent results within the same patient population [31].

### 4.4. TEBCO in Pregnancy

The unique physiological context of pregnancy amplifies the potential of TEBCO. Its noninvasive, radiation-free technology makes it especially suitable for maternal monitoring. While earlier reports suggested that TEB is most reliable for tracking relative changes rather than absolute values, recent studies indicate that it can also provide clinically meaningful insights into distinct hemodynamic profiles [19,39]. On the other hand, pregnancy-specific factors, including weight gain, breast tissue, and fluid shifts, may impair signal quality [40].

By enabling bedside, repeatable, and continuous monitoring of maternal hemodynamics, TEBCO may fill a critical gap in the management of HDP, particularly in settings where rapid, noninvasive assessment is essential for maternal and fetal safety.

## 5. Validation Studies in Pregnant Populations

### 5.1. Normotensive Pregnancies

Initial investigations of TEBCO in pregnancy sought to determine whether this noninvasive technology could reproduce established patterns of maternal cardiovascular adaptation. Myhrman P and colleagues conducted one of the earliest longitudinal studies in healthy gravidas and found that TEBCO reliably tracked the characteristic rise in output during early and mid-gestation, followed by stabilization toward term [40]. Although absolute values diverged slightly, the overall trajectory and magnitude of change were consistent.

Building on this early data, several studies assessed the reliability and reproducibility of modern impedance-based systems under physiologic conditions of pregnancy. Easterling et al. (1989) [37] evaluated the accuracy of impedance cardiography (ICG) for measuring cardiac output in pregnant women by comparing it directly with the thermodilution technique, the clinical gold standard. Their study demonstrated that although ICG tended to systematically underestimate absolute cardiac output values, the method showed consistent directional changes and acceptable correlation with thermodilution measurements [41]. In addition, Tomsin et al. (2012) [39] examined the reproducibility of noninvasive hemodynamic measurements obtained by impedance cardiography in 40 healthy pregnant women and evaluated the influence of posture. Their results confirmed high intra-observer reliability and minimal postural variation in key parameters such as cardiac output, systemic vascular resistance, and thoracic fluid content, reinforcing the robustness of TEBCO for serial assessments throughout gestation [39].

Complementing these validation studies, a systematic evaluation by Meah et al. (2016) [6] synthesized available data on cardiac output during normal pregnancy. Their meta-analysis, which included impedance-based and Doppler studies, confirmed the expected physiological pattern of increasing CO and declining SVR across gestation, lending strong support to the validity of TEBCO for trend monitoring [6]. More recent prospective cohorts by Vårtun et al. (2015) and Andreas et al. (2016) further confirmed that impedance-derived indices faithfully capture the progressive rise in CO, HR, and thoracic fluid content, accompanied by a fall in SVR, consistent with the physiologic adaptations of normal pregnancy [41,42].

Collectively, these investigations demonstrate that, while absolute values may vary between technologies, TEBCO and related impedance-based methods consistently reflect the dynamic cardiovascular adaptations of normal pregnancy and are well-suited for tracking relative changes over time.

### 5.2. Intrapartum Studies

While most evidence for TEBCO comes from antepartum settings, there is growing support for its use in the intrapartum period, especially during cesarean delivery. In 2005, Tihtonen et al. used whole-body impedance cardiography in elective cesarean sections under spinal anesthesia. They observed an approximately 47% increase in cardiac index and a 39% drop in SVR index within minutes of delivery, even though MAP remained relatively stable [43].

More recently, D’Ambrosio et al. (2018) carried out a randomized, double-blind study during cesarean section, comparing two doses of levobupivacaine, and showed that impedance cardiography could capture dose-dependent changes in SVR, CI, and related indices after anesthesia induction [44].

These studies demonstrate the sensitivity of TEBCO to rapid preload and afterload changes associated with surgical delivery and anesthetic interventions. Formal validation against invasive reference standards in obstetric intrapartum settings, however, remains rare, largely for ethical and logistical reasons. The existing data underscores the feasibility and potential clinical value of real-time monitoring during labor and cesarean, particularly for anticipating hemodynamic destabilization (e.g., hypotension with anesthesia, blood loss, or effects of uterotonics).

### 5.3. Postpartum Studies

Thoracic electrical bioimpedance provides a dynamic window into the cardiovascular transition that follows delivery. In healthy women, serial TEBCO measurements demonstrate the expected physiological reversal of gestational hypervolemia and vasodilation. CO and SV fall sharply within the first 24–48 h postpartum, while SVR rises, gradually returning toward nonpregnant levels over the ensuing months. In a longitudinal study that followed women from mid-pregnancy to 48 h, 2 months, and 6 months postpartum, San-Frutos et al. reported this biphasic pattern, an early hemodynamic contraction followed by slow normalization of SVR and CO [45]. In a complementary cohort including women with preeclampsia, the same group showed that recovery was delayed and heterogeneous among hypertensive patients, some of whom maintained a high-resistance, low-output profile well beyond the early puerperium [46]. These findings underscore TEBCO’s ability to characterize recovery trajectories rather than single-point measurements.

Additional impedance-based work has provided reference ranges and population-level expectations for the immediate postpartum period. Morris et al. established normative impedance cardiography data for the first 48 h after vaginal and cesarean deliveries, defining practical interpretive limits for CO, SV, and SVR in uncomplicated patients [47].

Beyond healthy populations, persistent postpartum abnormalities have been documented using complementary hemodynamic modalities. Melchiorre et al. demonstrated that women with preeclampsia often exhibit sustained elevations in SVR and depressed CO for weeks after delivery, consistent with incomplete vascular and cardiac recovery [48]. These findings reinforce the clinical relevance of TEBCO as a noninvasive, repeatable bedside tool to track hemodynamic normalization, identify women at risk for prolonged dysfunction, and potentially guide follow-up strategies for long-term cardiovascular prevention.

Overall, evidence from impedance and related hemodynamic studies highlights the postpartum period as a critical phase of cardiovascular adaptation rather than passive recovery. The ability of TEBCO to quantify directional trends in CO and SVR offers a practical and sensitive approach to monitor this transition. In both healthy and hypertensive women, it may provide early indicators of incomplete cardiovascular recovery, enabling closer follow-up and individualized management to mitigate future cardiovascular risk.

### 5.4. Studies in Hypertensive Disorders of Pregnancy

The validation of TEBCO in pregnancy began with feasibility studies and progressively expanded into hypertensive populations (Table 1). Masaki et al. (1989) first demonstrated that CO could be measured peripartum using impedance methods, including in women with hypertensive complications, confirming that the hypertensive state did not preclude reliable monitoring [49]. A decade later, Newman et al. (1999) [50] advanced this work by evaluating thoracic-fluid conductivity in women with preeclampsia. They showed that preeclampsia was associated with increased thoracic-fluid conductivity, stratified by disease severity, and identified that values ≥ 65 kΩ^−1^ were strongly associated with peripartum pulmonary edema [50]. Importantly, they suggested that women exceeding this threshold might be candidates for early medical intervention, even in the absence of overt symptoms, highlighting the role of impedance as a noninvasive predictor of pulmonary complications. Shortly thereafter, Scardo et al. (2000) validated TEBCO-derived cardiac output against M-mode echocardiography in preeclamptic women, reporting good agreement between the two methods and strengthening confidence in its clinical applicability [51].

Further studies characterized the hemodynamic heterogeneity of hypertensive disorders of pregnancy. San-Frutos et al. (2005) confirmed the classic pattern of reduced CO and elevated SVR in preeclampsia compared with normotensive pregnancies [46]. Laye et al. (2008) reported that elevated SVR index and thoracic fluid content were associated with severe and superimposed preeclampsia, suggesting that impedance parameters could help stratify severity [52]. Expanding into therapeutic applications, Chaffin et al. (2009) [53] retrospectively reviewed 318 singleton pregnancies at risk for hypertensive complications whose antihypertensive management was guided by impedance cardiography from <24 weeks’ gestation. All women received atenolol, and 24% required additional vasodilators based on hemodynamic profiles. They found that hyperdynamic patients, characterized by elevated CO, experienced lower rates of preeclampsia, less severe disease, fewer preterm deliveries <34 weeks, and fewer NICU admissions compared with those requiring vasodilators [53]. Overall, the study demonstrated that impedance-guided therapy allowed most pregnancies to reach term, with low rates of preeclampsia, minimal growth restriction, and no perinatal deaths. Parrish et al. (2012) [54] further refined diagnostic profiling, showing that patients with severe or superimposed preeclampsia were more likely to have depressed cardiac function and significantly higher BP, SVR, and thoracic fluid content compared with those with non-severe hypertensive disease. They concluded that impedance cardiography could rapidly and reliably identify women with severe or superimposed disease, supporting its role in risk stratification [54].

More recent work has emphasized longitudinal patterns and clinical outcomes. Andreas et al. (2016) established normative trajectories for CO and SVR during normal pregnancy and found that women at risk for preeclampsia or reduced birthweight demonstrated altered cardiovascular adaptation early in gestation [42]. Importantly, these distinct hemodynamic patterns could be detected by bioimpedance cardiography at low cost and without risk, suggesting potential for early identification of high-risk pregnancies. Hammad et al. (2019) extended the focus to pulmonary complications, reporting that thoracic fluid content correlated with the presence of pulmonary edema in preeclamptic women, reinforcing the use of TEBCO as a bedside tool for maternal safety [55]. In a large retrospective cohort of 958 women with chronic hypertension, Cottrell et al. (2022) demonstrated that impedance-guided antihypertensive therapy, using vasodilators for elevated resistance and beta-blockade for high cardiac output, was associated with reduced rates of fetal growth restriction and perinatal mortality, underscoring its potential to optimize outcomes in hypertensive pregnancies [56]. Most recently, Baztán Ilundain et al. (2024) [57] conducted a retrospective observational study of 55 pregnant and postpartum women with hypertensive disorders, analyzing cardiac work, CO, thoracic fluid content, and SVR indices. They reported positive correlations between systemic resistance and blood pressure, while fluid indices did not differ significantly between gestational hypertension and preeclampsia. Their results suggest that elevated SVR measured by TEBCO may serve as an early diagnostic marker of hypertensive disorders of pregnancy, though larger studies with normotensive controls are needed for validation [57]. Collectively, these investigations confirm the validity of TEBCO as a noninvasive modality capable of identifying disease-specific hemodynamic signatures, guiding antihypertensive therapy, and detecting pulmonary complications in hypertensive disorders of pregnancy.

### 5.5. Our Own Experience

We have evaluated six women with severe hypertensive disorders of pregnancy who underwent hemodynamic monitoring using the HOTMAN system (TEB-CO) to adjust medical treatments. Therapy was adjusted based on hemodynamic profiles, rather than empirical or guideline-driven medical therapies. Antihypertensive therapy was modified according to hemodynamic findings, including the addition of diuretics in one case. Clinical course and hypertension control were monitored until delivery. Student’s t test was used to compare MAP and SBP before (M1) and after (M2) treatment. The median gestational age (GA) at hemodynamic profiling was 34.2 weeks. Mean BMI was 40.8 kg/m2. 4/6 were primigravidas, 4/6 were already on antihypertensive treatment and 5/6 were managed as outpatients.

**Figure 3:**
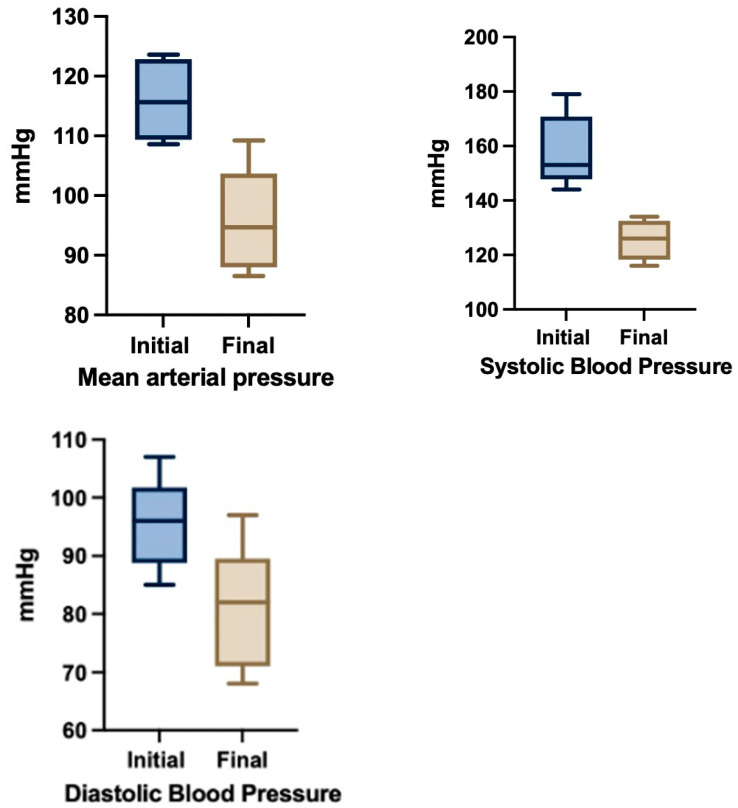
Blood pressure changes after TEBCO-drive optimization of hypertensive treatment (N = 6).

Symptomatology subsided in all cases after changes in therapeutic regimen. Statistically significant improvements of MAP (M1 = 116.0 to M2 = 95.9 mmHg, *p* = 0.0084) and SBP (M1 = 158 to M2 = 125.5 mmHg, *p* = 0.00027) were noted following treatment adjustments based on TEB-CO evaluation (Figure 3). The median GA at delivery was 36.93 weeks. There were no hypertension-related maternofetal morbidities. Importantly, no hypertension-related maternal or fetal morbidities were reported, and most patients were successfully managed as outpatients. Though the series is small, our experience supports the feasibility and potential utility of TEBCO-guided therapy in real world obstetric practice, highlighting its role in individualizing management strategies beyond conventional approaches and the potential for cost-savings by avoiding hospital admissions [58].

### 5.6. Limitations of Existing Studies

The potential of TEBCO in the management of hypertensive disorders remains to be demonstrated in larger series of patients.

Considering the cost and limited availability of this technology, we envision the creation of referral centers where the hemodynamic monitoring of pregnancies affected with hypertensive disorders could be evaluated, treated, and monitored in response to hemodynamic-driven data. Through a more rational and pathophysiological mechanism-driven therapy, these interventions could lead to significant prolongation of pregnancies that would have otherwise terminated early for the fear of complications associated with hypertensive crises or poor response to monotherapies based on guidelines, and not hemodynamic principles. Future studies addressing cost-effectiveness would be required to move this investigational technique into the clinical arena. Unlike echocardiography, this technology is easy to administer, and specific therapies can be implemented via protocols aimed at modifying inotropy, chronotropy, systemic vascular resistance and hypervolemia.

In addition, the role of volume in hypertensive disorders has been recognized and accepted, but its study and management have been neglected. The possibility of increasing or reducing intravascular and extracellular components using colloids or diuretics, as demonstrated in one of the cases we present, deserves further investigation.

## 6. Conclusions

For too long the management of hypertensive disorders of pregnancy has been empirical, driven by the fear of complications and litigation and the guidelines of management based on level III evidence. By providing more granular data, the implementation of noninvasive cardiography/TEBCO in the evaluation and management of hypertensive disorders of pregnancy can identify explicit pathophysiological mechanisms, resulting in a more rational diagnosis and classification of these disorders. By elucidating precise hemodynamic alterations, tailored treatments can be implemented and clinical responses monitored serially, with the possibility of preventing severe morbidity and mortality and optimizing perinatal outcomes.

By offering a noninvasive, repeatable, and bedside-ready tool, noninvasive cardiography holds promise in refining risk stratification, improving phenotypic classification, and supporting the development of individualized management strategies in preeclampsia and related disorders. The adjunctive use of noninvasive cardiography in pregnancies complicated by hypertensive disorders would represent an improvement over the status quo, where terminations of pregnancy are recommended for severe hypertension or where non-selective and non-specific monotherapies are prescribed regardless of pathophysiological mechanisms, resulting in potential harm to the mother and/or the unborn fetus.

## Figures and Tables

**Table 1:** Studies of Thoracic Electrical Bioimpedance (TEBCO) in Hypertensive Disorders of Pregnancy.

Author (Year)	Population	Study Design	Comparator	Main Findings	Clinical Implication
Masaki et al. (1989) [49]	Peripartum women, incl. hypertensive (N ≈ 20)	Observational feasibility	—	Demonstrated feasibility of cardiac output measurement using impedance methods in obstetric patients	Established proof of concept that TEBCO can be applied in pregnancy including hypertensive states
Newman et al. (1999) [50]	Peripartum women (N = 134)	Observational study	Thoracic fluid conductivity vs. clinical severity	Preeclampsia associated with increased thoracic fluid conductivity stratified by severity; values ≥ 65 kΩ^−1^ strongly linked to peripartum pulmonary edema	Suggested that women with elevated conductivity may require early intervention even without overt symptoms
Scardo et al. (2000) [51]	Women with preeclampsia (N = 15)	Validation study	M-mode echocardiography	Good agreement between impedance-derived and echocardiographic cardiac output	Supported use of TEBCO as a reliable hemodynamic monitor in preeclampsia
San-Frutos et al. (2005) [46]	Normotensive vs. preeclamptic pregnancies (N = 45)	Cross-sectional comparison	Normotensive controls	Confirmed reduced cardiac output and increased systemic vascular resistance in preeclampsia	Demonstrated ability of TEBCO to differentiate hemodynamic profiles between normal and hypertensive pregnancies
Laye et al. (2008) [52]	Women evaluated for acute or chronic hypertension (N = 129)	Abstract	—	Elevated systemic vascular resistance index and thoracic fluid content associated with severe and superimposed preeclampsia	Suggested thresholds of impedance indices may stratify risk of severe disease
Chaffin et al. (2009) [53]	Singleton pregnancies with chronic hypertension or prior preterm delivery due to preeclampsia (N = 318)	Retrospective cohort with impedance-guided therapy	Hemodynamic subsets compared; all received atenolol, 24% vasodilator	ICG-guided therapy associated with mean GA at delivery of 37 ± 2 weeks; PE incidence 14%; BW < 10th % in 10%; no perinatal deaths. Hyperdynamic patients had less severe PE, fewer preterm births, fewer NICU days	Showed that tailoring therapy by impedance profile optimizes maternal and neonatal outcomes
Parrish et al. (2012) [54]	Women with hypertensive disorders (N = 129)	Cross-sectional diagnostic study	Comparison among hypertensive subgroups	Severe/superimposed PE cases showed depressed cardiac function, higher BP, MAP, SVR, and TFC vs. non-severe hypertensive disease	ICG hemodynamic profiling rapidly identifies severe or superimposed PE, aiding risk stratification
Andreas et al. (2016) [42]	Healthy pregnancies (N = 242)	Longitudinal cohort	—	Documented normative trajectories of CO and SVR. Women at risk for PE or low birthweight showed altered adaptation patterns detectable early in pregnancy	Demonstrated that TEBCO can identify abnormal cardiovascular adaptation early at low cost and without risk
Hammad et al. (2019) [55]	Parturients with preeclampsia (N = 60)	Diagnostic comparison	Lung ultrasound	Thoracic fluid content correlated with pulmonary edema in PE patients	Supported role of TEBCO as a bedside tool for noninvasive detection of pulmonary complications
Cottrell et al. (2022) [56]	Women with chronic hypertension (N = 958)	Retrospective cohort with impedance-guided therapy	Therapy guided by CO and SVR indices	Vasodilators used for high SVR; beta-blockers for high CO. Associated with reduced FGR and perinatal mortality	Confirmed that ICG-directed therapy improves outcomes in chronic hypertension and is relevant to HDP care pathways
Baztán Ilundain et al. (2024) [57]	Pregnant and postpartum women with HDP (N = 55)	Retrospective observational study	Comparisons across HDP subtypes	Median LCWI 5.05 kg·m/m^2^; CO 6352 mL/min; TFCI 17.9 L/kΩ/m^2^; SVRI 2168 dyn·s·cm^−5^·m^2^. SVRI correlated with BP; no correlation between TFCI and BP; no significant differences between GH and PE groups	Suggested elevated SVR measured by TEBCO may serve as early diagnostic marker of HDP

CO = cardiac output; SVR = systemic vascular resistance; SVRI = systemic vascular resistance index; TFC = thoracic fluid content; TFCI = thoracic fluid content index; PE = preeclampsia; GH = gestational hypertension; FGR = fetal growth restriction; HDP = hypertensive disorders of pregnancy; GA = gestational age; BW = birth weight; MAP = mean arterial pressure; LCWI = left cardiac work index; NICU = neonatal intensive care unit.

## Data Availability

No new data were created or analyzed in this study. Data sharing is not applicable to this article.
